# Exploring a 1-Minute Paced Deep-Breathing Measurement of Heart Rate Variability as Part of a Workers’ Health Assessment

**DOI:** 10.1007/s10484-018-9422-4

**Published:** 2018-12-01

**Authors:** Marianne Six Dijkstra, Remko Soer, André Bieleman, Rollin McCraty, Frits Oosterveld, Douglas Gross, Michiel Reneman

**Affiliations:** 1 Saxion University of Applied Sciences/AGZ, M.H. Tromplaan 28, 7500 KB Enschede, The Netherlands; 2 Groningen Spine Center, University Medical Center Groningen, University of Groningen, Groningen, The Netherlands; 3 Heartmath LCC, Boulder Creek, CA USA; 4 Department of Physiotherapy, University of Alberta, Edmonton, Canada; 5 Department of Rehabilitation Medicine, University Medical Center Groningen, University of Groningen, Groningen, The Netherlands

**Keywords:** Employment, Workers, Screening, Heart rate variability, Diagnostic techniques and procedures

## Abstract

Low heart rate variability (HRV) is related to health problems that are known reasons for sick-leave or early retirement. A 1-minute-protocol could allow large scale HRV measurement for screening of health problems and, potentially, sustained employability. Our objectives were to explore the association of HRV with measures of health. Cross-sectional design with 877 Dutch employees assessed during a Workers’ Health Assessment. Personal and job characteristics, workability, psychological and mental problems, and lifestyle were measured with questionnaires. Biometry was measured (BMI, waist circumference, blood pressure, glucose, cholesterol). HRV was assessed with a 1-minute paced deep-breathing protocol and expressed as mean heart rate range (MHRR). A low MHRR indicates a higher health risk. Groups were classified age adjusted for HRV and compared. Spearman correlations between raw MHRR and the other measures were calculated. Significant univariable correlations (*p* < 0.05) were entered in a linear regression model to explore the multivariable association with MHRR. Age, years of employment, BMI and waist circumference differed significantly between HRV groups. Significant correlations were found between MHRR and age, workability, BMI, waist circumference, cholesterol, systolic and diastolic blood-pressure and reported physical activity and alcohol consumption. In the multivariable analyses 21.1% of variance was explained: a low HRV correlates with aging, higher BMI and higher levels of reported physically activity. HRV was significantly associated with age, measures of obesity (BMI, waist circumference), and with reported physical activity, which provides a first glance of the utility of a 1-minute paced deep-breathing HRV protocol as part of a comprehensive preventive Workers’ Health Assessment.

## Introduction

With an aging workforce (Douwes et al. 2014; Duin and Stoeldraijer 2013; Sas and Suarez 2014), the need increases to preserve health and wellbeing as employees work until retirement age. Ageing leads to an increased risk of developing chronic diseases (Sas and Suarez 2014). Chronic diseases in ageing workers are associated with decreased workability (Leijten et al. 2014) and health issues are the most common reason for leaving the workforce before the statutory retirement age (Perosh 2014). Workers with long sickness absence are more likely to exit the labor force through disability benefits and unemployment (Reeuwijk et al. 2015).

In the Netherlands, the main reasons for calling in sick longer than 3–4 weeks are psychological (33%) and musculoskeletal (28.9%) problems. In the group of ‘other diagnoses’ (38.1%), the main reasons for long term sick-leave are coronary heart disease (4.9%), intestinal (4.8%) and neurological (4.7%) problems (Volksgezondheidenzorg.info 2017). Between 2007 and 2013, the number of absent days due to concentration problems and fatigue increased. When duration of sick-leave is accounted for, musculoskeletal problems, psychological problems, including burnout and being overworked account for 40% of the total sick-leave volume. Early identification of these potentially modifiable health problems and prevention initiatives could decrease sick-leave and improve sustained employability (Leijten et al. 2014).

Workers’ Health Assessments (WHA) are used to identify specific work-related risks in order to prevent injuries or disease to promote sustained employability (WHO 2002; van Holland et al. 2015; Weel et al. 2007). A periodic medical examination can be part of a WHA (Aldana et al. 2005). Health status and work-related problems are often assessed with biometrical measurements and questionnaires. Biometrical measurements can be used to assess risk for metabolic syndrome, diabetes and coronary heart disease. Self-report measures often include the Work Ability Index (WAI), which aims specifically at determining workability (Ilmarinen 2009) and includes questions on neurological, intestinal and coronary heart diseases. A large number of other questionnaires may be used to assess the physical and mental health status and associated risks, as well as perceived work strain. All parameters together provide information about the health and sustained employability status of the employee. However, the construct of sustained employability is difficult to measure and more objective measures may increase the chances for finding workers with health-related threats to sustained employability.

One biometrical concept that may be related to sustained employability is heart rate variability (HRV). HRV is associated with physical and mental health issues (Beauchaine and Thayer 2015; Geisler et al. 2013; McCraty et al. 1998; Wulsin et al. 2015) and, in turn, potentially to sustained employability. HRV is a measure of the consecutive differences in time between heartbeats. The oscillation in the beat-to-beat interval of heart rate is the result of hormonal, neural and mechanical interactions of local and central systems (heart rate variability: standards of measurement, physiological interpretation and clinical use. Task Force of the European Society of Cardiology and the North American Society of Pacing and Electrophysiology 1996). HRV is a marker for functioning of the autonomic nervous system (Shaffer et al. 2014). The time variations between consecutive heartbeats in a healthy heart are large and reflect the ability of the brain, autonomic nervous system and heart to adapt to changing circumstances. HRV declines with age (Umetani et al. 1998), but lower HRV values are also an independent predictor of decreased health (Dekker et al. 2000). Low HRV is indicated as a predictor for metabolic syndrome and cardiovascular diseases (Dekker et al. 2000; Liao et al. 1998; Licht et al. 2013; Thayer and Lane 2007; Tsuji et al. 1996; Wulsin et al. 2016). Low HRV has been observed in patients with diabetes, hypertensive cardiac hypertrophy and atherosclerosis. Low HRV is also associated with lower self-regulation and resilience (Geisler et al. 2013), with lower mental and social flexibility (Geisler et al. 2013; Williams et al. 2015), was found to be a marker for fatigue (Volker et al. 2016), and is more present in people with clinical burnout compared to healthy controls (Lennartsson et al. 2016). Summarized, a low HRV for one’s age is related to several health issues that may compromise a worker’s sustained employability. There is, however, insufficient knowledge about the additional value of measuring HRV in a working population with the focus on sustained employability.

For HRV, 24-h measurements are considered the gold standard but are expensive and time consuming. However, new technologies now enable HRV measurement in the occupational setting as a potential health screen. With photo plethysmography (PPT) technology, rapid and non-invasive measurements became available. Promising results have been reported for a drastically shortened 1-minute paced deep-breathing protocol (Russoniello et al. 2013). The next step to validate HRV as a potential screening instrument for future SE will be to explore associations of HRV with health related issues. Therefore, the objectives of this study are to: (1) compare the WHA outcomes of workers with a very low, low and normal HRV; and (2) examine associations between a 1-minute HRV measurement and health related outcomes derived from a WHA.

## Methods

### Study Design and Setting

A cross-sectional design was carried out. In the Netherlands, employers of moderate or large companies are required to offer their workers a voluntary health assessment, performed by an independent occupational healthcare supplier (OHS). Data from WHAs that were part of a regular health screening service were collected between November 2015 and June 2016 and explored in relation to HRV. Three OHS’s performed the measurements examined in the current study. The Ethics Board at the University Medical Center Groningen in The Netherlands decided that formal approval of the study was not necessary because all workers were subjected to care as usual only.

### Participants

Workers from divisions of 14 companies in the Netherlands were included. All workers were invited to participate. The company was responsible for the invitation process. Data of workers between 18 and 65 years were used. Pregnant women and workers with severe health problems who were currently absent from work and workers with pacemakers were excluded from this study.

### Data Collection

The WHA data were collected using two procedures. The first was an online questionnaire completed at home (30–45 min) prior to assessment. The second procedure was a physical assessment that included biometric measurements collected by a physician assistant or occupational physical therapist (OPT) at the company (25 min). Immediately after the biometric measurements, there was a counseling session with an OPT (25 min). The time between answering the questionnaire and the biometry measurements varied between 2 weeks and 1 h. For HRV, raw data were collected during the test in the emWave® Pro Plus software (Heartmath Institute, Boulder Creek). The participants were asked not to eat or drink for 45 min prior to the appointment (which was 60 min prior to the HRV measurements), and to avoid heavy physical work during the last hour.

### Measurements

#### Questionnaires

Personal (age, sex, level of education) and work characteristics [working hours per week, number of years employed, workload [categories of the Dictionary of Occupational Titles (DOT)] were registered. In the DOT, occupations are classified into five categories of physical workload, based on intensity and duration of lifting or carrying needed for the job: sedentary, light, medium, heavy/very heavy. Because very heavy work hardly exists in the Netherlands, the last two categories were combined (Soer et al. 2009).

The following questionnaires were administered for the study:

1. Work Ability was measured with the short version of the Work Ability Index (WAI). It consists of seven items (Tuomi et al. 1998). The WAI has been shown to be valid for determination of sustained employability (Alavinia et al. 2008; de Zwart et al. 2002; Radkiewitz and Widerszal-Bazyl 2005) and is internally consistent [Cronbach’s alpha 0.72–0.80 (Tuomi et al. 2004)]. The scale ranges between 7 and 49, with higher scores indicating better workability. WAI can be used to identify workers at high risk for prolonged sickness absence (Reeuwijk et al. 2015).
2. Work engagement was measured with the Utrecht Work Engagement Scale-9 (UWES-9) (Schaufeli et al. 2006; Schaufeli and Bakker 2003). The UWES-9 consists of nine questions and assesses ‘work engagement’. It consists of three subscales, measuring dedication (three questions), vigor (three questions), and absorption (three questions), with a 0–6 point scale per question. The total scale ranges between 0 and 54, with a higher score indicating more engagement. Psychometric properties of the questionnaire are good (Schaufeli et al. 2006; Schaufeli and Bakker 2003).
3. Psychological problems were measured with the General Health Questionnaire-12 (GHQ-12) (Koeter and Ormel 1991; Sluiter and Hulshof 2013), consisting of 12 questions with 4 possible answers; 2 are valued positive (score = 0) and 2 negative (score = 1). The scale ranges between 0 and 12, with a lower score indicating less functional psychological problems.
4. Perceived Workload was measured with subscales of the Questionnaire on the Experience and Evaluation of work (QEEW, in Dutch: VBBA) (Veldhoven et al. 2002): need for recovery (NfR, 11 items)—Workpace (Wp, 11 items)—perceived mental strain (MS, 9 items). Subscale scores range between 0 and 100, and a lower score indicates more favorable situations. Reliability is satisfactory (Veldhoven et al. 2002).
5. A lifestyle questionnaire asking about physical activity, smoking, alcohol, nutrition and recreation. The questionnaire is descriptive in nature, based on guidelines (RIVM 2016), and has not been validated as an outcome measure. The results are scored with an ordinal scale 1–3, with a lower score indicating healthier behavior.

Detailed information about the full protocol and interpretation of results is based on guidelines as presented in Appendix 1.

#### Heart Rate Variability

HRV was measured with the ear lobe pulse sensor of the emWave® Pro Plus. The emWave® Pro Plus uses photo pletysmography (PPG) technology, which is based on the ability of hemoglobin to absorb light and can measure the blood pulse wave through the skin. PPG technology is a reliable and valid method of capturing and quantifying HRV from a deep breathing test (Russoniello et al. 2013). The HRV measurement was recorded individually in a quiet room, in a slightly reclined (~ 10°) seated position. The sensor was placed on the earlobe and the participant was introduced to the emWave® Pro Plus software. Participants were instructed to remain seated and relaxed and to refrain from making any significant or rapid body movements. Participants were instructed to breathe according to the 1-minute, 6-breath protocol that was paced at a rhythm of 6 breaths per minute (0.1 Hz) while breathing as deeply as they comfortably could. This breathing method was designed to provide a physiological challenge to assess the maximum HRV range (amplitude) their system was capable of producing at that time (Shields 2009). A visual breath-pacer is used to facilitate the regularity of the breathing. The entire minute should be artefact-free. Compliance was closely monitored to ensure sufficient deep breathing and synchronization with the breath pacer. If quality was doubted, a second test was performed.

HRV is usually expressed by either time- or frequency-domain parameters (heart rate variability: standards of measurement, physiological interpretation and clinical use. Task Force of the European Society of Cardiology and the North American Society of Pacing and Electrophysiology 1996). For exploring the added value of HRV for a WHA, the mean heart rate range (MHRR) was used as outcome. The MHRR is a time domain variable indicating the magnitude of the amplitudes in acceleration and deceleration of the heart. The range of the interbeat intervals is measured and expressed in the average change in beats per minute (BPM). For each breathing cycle, the maximum in BPM is calculated and averaged over the 6 breaths to obtain the MHHR. MHRR has been suggested as a good indicator of autonomic nervous system health and biological age (Russoniello et al. 2013; Shields 2009) with the practical advantage that it can be explained easily to participants. Pearson correlation between MHRR and another regularly used measure, Root Mean Square of Successive Differences (RMSSD), was calculated to confirm the correlation between MHRR and RMSSD in this study compared to other studies.

#### Biometric Measures

Besides HRV, other biometric measures were: weight (kg), body length (m), waist circumference (cm), systolic and diastolic blood pressure (mmHg), glucose (mmol/l), and total cholesterol (mmol/l). BMI was also calculated (weight/length^2^) (see Appendix 1 for full protocol and cut off values).

### Data Handling

The EmWave Pro Plus® software calculated MHRR. All data from the questionnaires, biometry, and HRV were entered and processed in a software program from the OHS. All WHA data were compiled into an Excel file and de-identified by the OHS. The data were then sent to the researchers. Only data with a complete set for WHA and HRV were used for statistical analyses. For descriptives, the data were divided into subgroups because literature indicates low HRV is related to health problems (Dekker et al. 2000; Geisler et al. 2013; Lennartsson et al. 2016; Liao et al. 1998; Licht et al. 2013; Shaffer et al. 2014; Thayer and Lane 2007; Tsuji et al. 1996; Volker et al. 2016; Williams et al. 2015; Wulsin et al. 2016). In absence of validated meaningful cut-off values, statistical criteria were used to determine cut-off values. Cut-off values are provided on the left side of the normal distribution of MHRR of a healthy reference group (proprietary, Emwave Pro Plus® software): very low HRV when MHRR > 2 SD below mean, low HRV when MHRR is 1–2 SD below mean, normal-high HRV when the deviation is higher than 1 SD below mean. Categorization of HRV was age corrected, because most variance of HRV is explained by age and we are interested in the contribution of other measures.

### Statistical Analyses

First, to obtain insight into which levels of the WHA measures were observed in the workers with very low, low or normal-high HRV, descriptive results are presented for each group. Age was normally distributed and described with mean and standard deviation. The other personal and work characteristics and WHA data are provided with medians and minimum–maximum for ordinal and for skewed continuous data, frequencies and percentages for nominal data and the lifestyle-questionnaires data (because they have only three categories). Secondly, differences between groups were tested. An Anova was used for age. The other continuous variables were not normally distributed and therefore the non-parametric Kruskall–Wallis *H* test was used. Then all single associations between raw MHRR and raw WHA measures were calculated with a Spearman correlation. The 95% confidence interval (CI) around *r* was calculated with a bootstrap accelerated procedure (1000 ×). If the 95% CI did not cross zero, the association between MHRR and the WHA measure was considered significant. Finally a multivariable analysis was performed to explore the shared variance of combined measures (independent variables) to MHRR (dependent variable). The WHA measures with a significant single correlation with MHRR were included as independent measures for backward regression analyses. Continuous skewed data were 10log-transformed to meet the assumptions for linear statistics and dummy variables were made for categorical data. A best fit was found with the independent measures that contributed significantly to the model (*p* < 0.05). Adjusted R^2^ was calculated for cross-validation of the model. All results were statistically analyzed with IBM SPSS Statistics 24.0 software.

## Results

### Participants

Participation rate was 70–100% in the 14 included companies. 1420 Workers participated in the WHA; 155 were excluded from the study because they did not meet the inclusion criteria and 388 had incomplete WHA data, resulting in 877 participants included in the study. The majority of participants were male (77%), mean age was 43.4 years (*SD* = 10.2, range = 19–65). Most participants (44.0%) performed work with moderate physical demands (DOT3) and had a high education level (49.3%). Age (*F*(2,874) = 12.39, *p* = 0.00) and years of employment (*H*(2) = 8.87, *p* = 0.01) differed significantly between HRV groups; the post hoc Bonferroni and Mann–Whitney *U* test revealed that the group with normal-high MHRR was significantly younger than the other two groups and employed shorter than the low MHRR group. Characteristics of participants and work are presented in Table 1, divided by HRV (MHRR) category.

**Table 1:** Participants and work characteristics, divided by heart rate variability (MHRR) outcome

	Normal to high MHRR n = 794	Low MHRR n = 48	Very low MHRR n = 35	Total group n = 877
Age in years, mean (SD)*	43 (10)	47 (8)	50 (10)	43 (10)
Gender, n (%)				
Male	608 (76.6)	40 (83.3)	27 (77.1)	675 (77.0)
Female	186 (23.4)	8 (16.7)	8 (22.9)	202 (23.0)
Education level, n (%)				
Very low	24 (3.0)	1 (2.1)	1 (2.9)	26 (0.3)
Low	91 (11.5)	9 (18.8)	7 (20)	107 (12.2)
Intermediate	278 (35.1)	18 (37.6)	15 (42.9)	311 (35.4)
High	400 (50.4)	20 (41.6)	12 (34.3)	432 (49.3)
DOT category, n (%)				
DOT1	92 (31.5)	6 (35.3)	7 (43.8)	105 (32.0)
DOT2	65 (22.3)	7 (41.2)	3 (18.8)	75 (23.1)
DOT3	133 (45.5)	4 (23.5)	6 (37.5)	143 (44.0)
DOT4/5	2 (0.7)	0 (0)	0 (0)	2 (0.0)
Irregular shifts, n (%)				
Yes	604 (76.2)	37 (77.1)	25 (71.4)	666 (75.9)
No	189 (23.8)	11 (22.9)	10 (28.6)	210 (23.9)
Contract, n (%)				
Fixed	633 (90.3)	39 (92.9)	27 (96.4)	699 (79.7)
Temporary	68 (9.7)	3 (7.1)	1 (3.6)	72 (8.2)
Working hours/ week, median (min–max)	40 (4–48)	40 (20–48)	38 (1–56)	40 (1–56)
Years employed, median (min–max)*	5 (0–48)	6 (1–39)	8 (1–46)	5 (0–48)

Level of education: very low = no or elementary education; low = lower vocational education; intermediate = intermediate vocational education and secondary higher level education; high = bachelor or higher education; DOT = dictionary of occupational titles. Three descriptives were not applied by everyone: DOT category, n = 325; irregular shifts, n = 876; contract, n = 871

*Groups differ significantly with p ≤ 0.05

### WHA Results

The WHA measures are presented in Table 2, divided by HRV category.

**Table 2:** Results of the Workers Health Assessment (WHA) measures, divided by heart rate variability (MHRR) outcome

	Normal-high MHRR n = 794 Median (min–max)	Low MHRR n = 48 Median (min–max)	Very low MHRR n = 35 Median (min–max)	Total group n = 877 Median (min–max)
MHRR (beats/min)*	21.0 (7.7–66.6)	8.0 (6–11)	5.3 (2–9)	19.8 (2–66.6)
BP Diastolic (mmHg)	80.0 (54–120)	80.0 (65–107)	81.0 (65–103)	80.0 (54–120)
BP Systolic (mmHg)	132.0 (96–205)	135.0 (104–180)	136.0 (110–185)	132.0 (96–205)
Cholesterol (mmol/l)	5.2 (2.6–9.3)	5.3 (3.1–7.7)	5.3 (3.6–7.8)	5.1 (2.6–9.3)
Glucose (mmol/l)	4.8 (1.2–16.8)	4.7 (2.7–12.7)	4.7 (2.6–7.2)	4.8 (1.2–16.8)
BMI (kg/m^2)^*	25.4 (17.5–42.6)	26.6 (19.8–37.9)	26.3 (18.4–41.3)	25.5 (17.5–42.6)
Waist circumf. (cm)*	92.0 (59–140)	96 (77–131)	95 (71–127)	93 (59–140)
WAI	43.0 (25–49)	41.5 (31–49)	44.0 (19–49)	43.0 (19–49)
UWES-9	39.0 (17–45)	40.5 (14–45)	37.0 (5–45)	38.7 (5–54)
GHQ-12	0.0 (0–7)	0.5 (0–10)	1.0 (0–12)	0.0 (0–12)
QEEW-NfR	18.2 (0–64)	9.1 (0–91)	18.2 (0–91)	18.2 (0–91)
QEEW-Wp	33.3 (18–67)	30.3 (9–58)	36.4 (0–100)	33.3 (0–100)
QEEW-MS	77.8 (44–89)	77.8 (33–89)	77.8 (0–100)	77.8 (0–100)

	Normal-high MHRR n = 794 n (percentage)	Low MHRR n = 48 n (percentage)	Very low MHRR n = 35 n (percentage)	Total group n = 877 n (percentage)
Physical activity^a^				
Normal risk	307 (38.7)	20 (41.7)	17 (48.6)	344 (39.2)
Intermediate risk	63 (7.9)	3 (6.3)	2 (5.7)	68 (7.8)
High risk	424 (53.4)	25 (52.1)	16 (45.7)	465 (53.0)
Smoking^a^				
Normal risk	647 (81.5)	41 (85.4)	28 (80)	716 (81.6)
Intermediate risk	16 (2)	1 (2.1)	1 (2.9)	18 (2.1)
High risk	131 (16.5)	6 (12.5)	6 (17.1)	143 (16.3)
Alcohol^a^				
Normal risk	697 (87.8)	39 (81.3)	30 (85.7)	766 (87.3)
Intermediate risk	81 (10.2)	7 (14.6)	4 (11.4)	92 (10.5)
High risk	16 (2.0)	2 (4.2)	1 (2.9)	19 (2.2)
Nutrition^a^				
Normal risk	436 (54.9)	24 (50)	21 (60)	481 (54.8)
Intermediate risk	86 (10.8)	4 (8.3)	11 (31.4)	101 (11.5)
High risk	272 (34.3)	20 (41.7)	3 (8.6)	295 (33.6)
Recreation^a^				
Normal risk	388 (48.9)	30 (62.5)	17 (48.6)	435 (49.6)
Intermediate risk	274 (34.5)	14 (29.2)	13 (37.1)	301 (34.3)
High risk	132 (16.6)	4 (8.3)	5 (14.3)	141 (16.1)

*MHRR* mean heart rate range, *WAI* Work Ability Index-short version, scale 7–49, *UWES-9* Utrecht Work Engagement Scale-9 questions (scale 0–54), *GHQ-12* General Health Questionnaire (measure psychological problems)-12 questions (scale 0–12), *QEEW* Questionnaire on the Experienced and Evaluation of Work (scale 0–100), *NfR* need for recovery, *Wp* workpace, *MS* mental strain

*Groups differ significantly with p ≤ 0.05

^a^‘Lifestyle-risk’ indicates the risk of health problems due to unhealthy behavior

#### Mean Heart Rate Range

In the studied population, when controlled for age 4.0% of the workers had a very low HRV, 5.5% a low HRV, and 90.5% had a normal-high HRV (Table 2).

#### Questionnaires

Median workability was good (> 41) in all groups. All groups reported being engaged, had few problems regarding need for recovery, psychological problems, or work pace. Mental strain was reported high in all groups. Differences between groups are small and insignificant with large variations within groups. Observation of the lifestyle results show that in the group with normal-high HRV, 53.4% were not physically active enough, while in the group with very low HRV this was 45.7%. Alcohol consumption and smoking were reported as a normal risk by most of the workers (more than 80% in all groups) and only 2.2% were classified in the high risk alcohol category. For the statistical comparison the moderate and high health risk of lifestyle factors were collapsed, to ensure cells were filled with more than five participants. Although some small differences in lifestyle were observed, these were statistically insignificant (*H(2)* = 3.37, *p* = 0.20 (recreation) to *H*(2) = 0.53, *p* = 0.79 (smoking)), including physical activity (*H*(2) = 1.51, *p* = 0.48).

#### Biometry

Differences in biometry between groups were small. BMI was significantly different (*H*(2) = 7.22, *p* = 0.03) and waist circumference was borderline significantly different (*H*(2) = 5.96, *p* = 0.05) between groups. A post hoc Mann–Whitney U test revealed that only the very low HRV group (*median BMI* = 26.3) differed significantly from the normal-high HRV (*median BMI* = 25.4) group for BMI (*U* = 11140.5, *z* = − 1.99, *p* = 0.047). All groups on average were overweight.

### Correlations

The continuous parameters were skewed and a non-parametric Spearman test was used. Correlation between RMSSD and MHRR was *r* = 0.66 (*p* < 0.01). Because the residuals did not meet the assumptions of homoscedasticity, the 95% CI around *r* was created with a robust Bias-Corrected and accelerated bootstrap procedure of 1000 samples (BCa 95%). The Spearman correlation was small but significant for most of the biometry measures (diastolic and systolic blood-pressure, cholesterol, BMI, waist circumference), the workability index, physical activity, and alcohol consumption, but not for the other measures (Table 3). MHRR decreases when more physical activity is reported, the association with alcohol was negative. In line with previous literature (Almeida-Santos et al. 2016; Koenig et al. 2015), age and MHRR correlated *r* = 0.40 (*p* < 0.01) in our study, which confirms the necessity to control for age in the regression analyses (Table 4).

**Table 3:** The correlation of raw MHRR with parameters of the WHA

	Spearman’s correlation		
	R	SE	BCa 95% (lower, upper)
Age	− 0.44^a^	0.03	− 0.50, − 0.38
Blood-pressure. systolic	− 0.22^a^	0.03	− 0.28, − 0.15
Blood-pressure. diastolic	− 0.17^a^	0.03	− 0.23, − 0.10
Cholesterol	− 0.18^a^	0.03	− 0.24, − 0.12
Glucose	0.02	0.03	− 0.05, 0.09
BMI	− 0.20^a^	0.03	− 0.26, − 0.16
Waist circumference	− 0.21^a^	0.03	− 0.27, − 0.15
WAI-short	0.13^a^	0.03	0.07, 0.20
UBES-9	0.01	0.04	− 0.06, 0.08
GHQ-12	− 0.00	0.03	− 0.07, 0.07
QEEW-need for recovery	0.00	0.04	− 0.06, 0.07
QEEW-workpace	0.04	0.04	− 0.03, 0.11
QEEW-mental strain	0.00	0.03	− 0.06, 0.07
Physical activity	0.08^a^	0.03	0.01, 0.14
Smoking	− 0.05	0.03	− 0.12, 0.02
Alcohol	− 0.10^a^	0.04	− 0.16, − 0.02
Nutrition	0.03	0.03	− 0.04, 0.10
Recreation	0.04	0.03	− 0.03, 0.11

The 95% confidence interval is calculated with a bias-corrected and accelerated (BCa) bootstrap procedure (1000 samples)

*R* correlation coefficient, *SE* standard error, *BMI* Body Mass Index, *BCa* bias-corrected and accelerated bootstrap, *MHRR* mean heart rate range, *WAI-short* Work Ability Index-short version, *UWES-9* Utrecht Work Engagement Scale-9 questions, *GHQ-12* General Health Questionnaire (measure: psychological problems)-12 questions, *QEEW* Questionnaire on the Experienced and Evaluation of Work

^a^BCa 95% does not cross 0, indicating a significant correlation

**Table 4:** Results of the linear regression analyses with a backward procedure

Dependent variable: 10log (MHRR)	Unstandardized B	Standardized B	BCa 95.0% for B		SE	Sig.	VIF
			Lower bound	Upper bound			
Constant	2.28		1.95	2.60	0.16	0.00	
Age	− 0.009	− 0.418	− 0.011	− 0.008	0.001	0.00	1.04
10log (BMI)	− 0.45	− 0.11	− 0.67	− 0.21	0.12	0.00	1.05
Physical activity	0.04	0.08	0.01	0.07	0.01	0.01	1.01

*MHRR* mean heart rate range, *Unstandardized B* regression coefficient, *SE* standard error, *Standardized B* standardized regression coefficient, *BMI* Body Mass Index, *BCa* bias-corrected and accelerated bootstrap, *VIF* = variance inflation factor

The 95% confidence interval around B is calculated with a Bias-corrected and accelerated (BCa) bootstrap procedure (1000 samples). The final model with 3 independent parameters is presented. R^2^ = 21.1%, Adjusted R^2^ = 20.8% *F*(3, 876) = 77.90, p ≤ 0.01

Physical activity coding: 0 = healthy physical active behavior, 1 = moderate or unhealthy physical active behavior

### Regression Analyses

BMI, waist circumference, blood-pressure (systolic and diastolic), cholesterol, WAI, physical activity and alcohol consumption were entered in the linear regression model with the BCa 95% procedure, controlled for age. MHRR and biometry were 10log-transformed. The WAI was treated as a continuous scale because dummy variables would be very uninterpretable with the wide scoring range (7–49) of the WAI. Reversed WAI data were 10log-transformed.

In the first model the nine variables are not significantly associated with HRV when entered together. BMI and waist-circumference both showed VIF values greater than 3 and systolic and diastolic blood-pressure showed VIF values greater than 2, indicating collinearity. With the backward procedure, in each step the least significant variable is excluded from the model until a model is found with only significant contributing variables and acceptable collinearity. Appendix 2 shows all steps of the backward procedure.

In the final model (adjusted R^2^ = 20.8%) with only significant contributing variables, 21.1% of MHRR was explained by age, BMI and Physical activity. Physical activity was coded as zero for healthy physical activity behavior and one for moderate and unhealthy physical activity behavior. BMI appeared to be a better predictor for MHRR than waist circumference.

In the final model, MHRR was significantly lower in aging workers with a higher BMI and healthy physical behavior.

## Discussion

The aim of this study was to explore associations between a novel compressed measure of HRV and other health related parameters, as a first step to studying the added value of HRV in a WHA. In this cross-sectional study, the very low HRV group was older and longer employed than the high HRV group. Because the classification was age corrected, a difference in age was remarkable. Although a forced breathing protocol was found to be a reliable and sensitive measure previously (Russoniello et al. 2013; Shields 2009), more studies in the occupational setting could contribute to stronger evidence for HRV reference values for age. Analyzing the raw MHRR data revealed significant univariate correlations between HRV and age, workability, blood pressure, cholesterol, BMI, waist circumference, physical activity and alcohol consumption. A high HRV was correlated to preferable health outcomes, except for physical activity. These results appear quite consistent with others (Almeida-Santos et al. 2016; Koenig et al. 2015) who also reported similar relations between HRV, age, and being overweight, although the strength of the associations may vary and the most dominant factors may differ [e.g. BMI in our study and waist circumference in others (Koenig et al. 2015)]. The result that HRV was lower when workers reported to be more physically active, controlled for age, was not expected. Others have reported that physical training had no or a positive effect on HRV on the long term (Amano et al. 2001; Jurca et al. 2004; Loimaala et al. 2000). In previous intervention studies (Amano et al. 2001; Jurca et al. 2004; Loimaala et al. 2000), exercise level was strictly controlled and, therefore, internal validity of these measurements is likely higher than internal validity of the self-report questionnaire in our study; Participants at risk may have provided preferable responses to our questionnaire regarding physical activity, biasing results. However, the observed association with physical activity was borderline significant and small and clinical relevance is questionable.

No significant association was observed between mental and psychological problems and MHRR. This result is not supported by others (Beauchaine and Thayer 2015; Geisler et al. 2013; Lennartsson et al. 2016; Thayer et al. 2009; Williams et al. 2015; Zahn et al. 2016), and might be attributed to floor and ceiling effects. In the current practical setting where predominantly healthy workers were assessed and prevalence of dysfunction in all the measured constructs was low, except for mental strain. This questionnaire however was not discriminative, since most workers scored very high on experienced mental strain (ceiling effect). With low prevalence and little variation, a significant association is less likely to be detected (Altman 1991).

We used MHRR as a measure for HRV because it is easier to explain to workers than the abstract time domain parameter RMSSD. However, RMSSD has been studied in most previous research examining correlations with psychological and mental problems (Beaumont et al. 2012; Geisler et al. 2013; Thayer et al. 2009; Williams et al. 2015; Zahn et al. 2016). In the current study, the correlation between RMSSD and MHRR was high (r = 0.66) and similar to other studies. The choice of MHRR as the HRV measure, therefore, is not expected to account for our lack of correlations observed. A post hoc study with RMSSD as dependent variable confirmed this assumption. To our knowledge this is only the second study (Russoniello et al. 2013) to examine MHRR assessed by a 1-minute deep paced-breathing protocol, and the first study in the context of WHA. More research would strengthen any conclusions.

The positioning of the lifestyle measures was different from the other measures. An unhealthy lifestyle is thought to precede health problems, while the other measures are already signs of a health risk. When a person has an unhealthy lifestyle, it is possible that the influence on the body and the autonomic nervous system is not yet measurable. Although we used the best available lifestyle questionnaires, these are not validated which limits our ability to draw strong conclusions. For example, reported alcohol consumption seemed to be very low in our high risk group (2.2%), compared to previous studies of the general population [7.3% (female)–10.4% (male) heavy drinkers in the Netherlands in 2016 (RIVM 2017)]. This again could indicate preferable answering by our participants.

Although the cut-off values were in line with the finding in previous literature that a low HRV level is related to health risks (Dekker et al. 2000; Geisler et al. 2013; Lennartsson et al. 2016; Liao et al. 1998; Licht et al. 2013; Shaffer et al. 2014; Thayer and Lane 2007; Tsuji et al. 1996; Volker et al. 2016; Williams et al. 2015; Wulsin et al. 2016), the estimated cut-off values were not based on meaningful diagnostic outcomes. The protocol used is relatively new and the necessary data are not available for sustained employability yet. We used the categorization to describe group characteristics, which might have been different with other cut-off values based on ROC curves. The categorization was not used in correlation and multivariable analyses, and had, therefore, no influence on the main study results.

A general point of discussion is that we compared subjective self-report measures with the objective measure, HRV. Although others (Jarczok et al. 2015) reported a relationship between HRV and self-reported health, this was not confirmed in the current study. Previous research on other health and functioning aspects (Bieleman et al. 2009; Reneman et al. 2002) has shown that self-report and physical measures do not correlate well, even when they aim to measure the same construct. This would support the idea that HRV (MHRR) could measure a different aspect of risk for psychological and mental health than the questionnaires. A longitudinal design should be applied to study the value of HRV as a screener for health problems and sustained employability.

This is the first study to explore a 1-minute paced deep-breathing HRV protocol as an objective screening measure for multiple future health issues in a working population. A large sample was included, which enhances external validity. The WHA protocol content was based on expert knowledge but limited by practical restrictions such as time constraints within the OHS encounters. Although from a theoretical point of view this might not be ideal, it can be argued that this is the way it works in actual practice and our design contributes to external validity of results. On the other hand, selection bias cannot be ruled out. The employers invited the participants to the WHA. Participation rate was supplied by the OHS and non-response numbers were not available. In this study, the focus was limited to finding cross sectional relationships and therefore the predictive value of HRV for future health had to be studied in relation to other predictors, and not to an outcome of future health. Since we studied an apparently healthy population, low prevalence of actual health problems might account for the low correlations observed. With a longitudinal study and a health behavior outcome measure (sick-leave or sustained employability), the predictive value of HRV in a WHA can be studied and cut-off values could be determined for this particular goal.

In conclusion, this study with apparently healthy workers showed that the age controlled, very low HRV group was significantly older, employed longer, less educated, had a higher BMI and larger waist circumference than the group with normal-high HRV. A lower HRV was significantly associated with aging, higher measures of obesity (BMI), and with higher levels of reported physical activity, which provides a first glance of the utility of a 1-minute paced deep-breathing HRV protocol as part of a comprehensive preventive Workers’ Health Assessment. Predictive validity of MHRR should be evaluated longitudinally as part of a screen for actual health outcomes. Based on this cross-sectional study of the value of HRV assessed by a 1-minute paced deep-breathing protocol, caution has to be taken for individual decision making when using only MHRR as a screen for future health and sustained employability.

## Appendix 1

See Table 5.

**Table 5:** Cut off values of the questionnaires, which are described in the methods section of the text

Cut off points	Not at risk (0 points)	Moderate risk (1 point)	High risk (2 points)
Activity	At least 5 days per week for 30 min low intensity activity AND at least 2 days per week 20 min. intensive active	At least 5 days a week 30 min. low intensity activity OR At least 2 days per week 20 min. intensive active	Less than 5 days per week 30 min. low intensity activity AND Less than 2 days per week 20 min. intensive active
Smoking	Non-smoker	≤ 2 days per week in combinations with ≤ 10 cigarettes per day	≥ 3 days per week or ≤ 2 days per week in combinations with ≥ 10 cigarettes per day
Alcohol	≤ 5 days per week and ≤ 15 drinks per week	> 5 days per week and ≤ 15 drinks per week OR ≤ 5 days per week > 15 drinks per week	> 5 days per week and > 15 drinks per week
Nutrition	Nutrition behavior without undesirable attitude as mentioned at moderate and high risk	≤ 4 days per week 3 meals or ≤ 4 days per week 200 gr vegetables	≤ 1 day, week 3 meals and/or ≤ 1 day, week 200 g vegetables and/or ≤ 4 days, week 3 meals plus ≤ 4 days, week 200 g vegetables
Recreation	Higher score = higher risk score is proprietary for OHS		
UWES-9	26–54	16–25	0–15
WAI-short	37–49	28–36	7 − 27
GHQ-12	0–3		> 3
QEEW-NfR	Scale 1–100: higher score = higher risk		
QEEW-Wp	Scale 1–100: higher score = higher risk		
QEEW-MS	Scale 1–100: higher score = higher risk		

*WAI* Work Ability Index-short version, *UWES-9* Utrecht Work Engagement Scale-9 questions, *GHQ-12* General Health Questionnaire (measure psychological problems)-12 questions, *QEEW* Questionnaire on the Experienced and Evaluation of Work, *NfR* need for recovery (11 questions), *Wp* workpace (11 questions), *MS* mental strain (9 questions)

### Biometry

Measurement information

- HRV: see article text.
- BMI: bodyweight (kg)/bodylength^2^ (m)
- Waist circumference: Participant stands upright, measure is taken of waist circumference at bellybutton level (in cm) while participant breathes out normally.
- Cholesterol: finger-stick total cholesterol measurement. In mmol per l.
- Glucose: finger-stick glucose measurement. In mmol per l.
- Blood pressure: participant is instructed not to perform heavy work at least 45 min prior to the measurement. Participant is sitting in slightly reclined position. Blood pressure is measured with a non-invasive automated measurement device.

See Table 6.

**Table 6:** Cutoff values of biometry

Normative values	Not at risk	Moderate risk	High risk
BMI	≤ 25.0	25.1–29.9	≥ 30.0
Waist circumference			
Female	< 80 cm	80–88 cm	> 88 cm
Male	< 94 cm	94–102 cm	> 102 cm
Cholesterol	< 5	5, 0–7, 8	< 7, 8
Glucose	< 7, 8	7, 8–11	> 11
Systolic blood pressure			
18–60 years	< 140	140–160	> 160
> 60 years	< 145	145–160	> 160
Diastolic blood pressure	< 90	90–100	> 100

*BMI* Body Mass Index

## Appendix 2

See Table 7.

**Table 7:** Results of the linear regression analyses with a backward procedure; MHRR is the dependent variable and nine variables of the workers health assessment are the independent variables

Dependent variable: 10log (MHRR)	Unstandardized B	Standardized B	BCa 95.0% for B		SE	Sig.	VIF
			Lower bound	Upper bound			
Step 1 (nine independent variables): R^2^ = 21.7%, adjusted R^2^ = 20.7%, F(11,865) = 21.78, p ≤ 0.01							
Constant*	2.22		1.53	2.92	0.36	0.00	
Age*	− 0.009	− 0.399	− 0.011	− 0.007	0.001	0.00	1.33
10log (BMI)*	− 0.53	− 0.14	− 0.97	− 0.11	0.23	0.02	3.60
Dummy1_PhysAct	0.02	0.05	− 0.03	0.08	0.03	0.39	3.74
Dummy2_PhysAct	0.02	0.04	− 0.03	0.07	0.03	0.44	3.77
Dummy1_Alc	− 0.02	− 0.03	− 0.06	0.03	0.02	0.45	1.22
Dummy2_Alc	0.01	0.01	− 0.12	0.15	0.07	0.81	1.20
10log (blood pressure diastolic)	− 0.21	− 0.05	− 0.56	0.13	0.18	0.23	2.09
10log (blood pressure systolic)	0.13	0.03	− 0.20	0.47	0.17	0.44	2.01
10log (waist circumference)	0.20	0.05	− 0.30	0.65	0.25	0.44	3.81
10log (cholesterol)	− 0.10	− 0.04	− 0.25	0.08	0.08	0.23	1.15
10log (reversed WAI + 1)	− 0.03	− 0.04	− 0.08	0.02	0.03	0.23	1.09
Step 2 (Alcohol_dummy2 excluded): R^2^ = 21.7%, adjusted R^2^ = 20.8%, F(10,866) = 23.98, p ≤ 0.01							
Constant*	2.21		1.51	2.95	0.36	0.00	
Age*	− 0.009	− 0.399	− 0.010	− 0.007	0.001	0.00	1.33
10log (BMI)*	− 0.54	− 0.14	− 1.02	− 0.10	0.23	0.03	3.58
Dummy1_PhysAct	0.02	0.05	− 0.03	0.07	0.03	0.33	3.71
Dummy2_PhysAct	0.02	0.04	− 0.03	0.07	0.02	0.45	3.75
Dummy1_Alc	− 0.02	− 0.02	− 0.06	0.03	0.02	0.52	1.05
10log (blood pressure diastolic)	− 0.21	− 0.05	− 0.56	0.14	0.18	0.24	2.09
10log (blood pressure systolic)	0.13	0.03	− 0.19	0.44	0.16	0.45	2.01
10log (waist circumference)	0.21	0.05	− 0.29	0.74	0.26	0.43	3.79
10log (cholesterol)	− 0.10	− 0.04	− 0.26	0.07	0.08	0.26	1.15
10log (reversed WAI + 1)	− 0.03	− 0.04	− 0.08	0.02	0.02	0.20	1.09
Step 3 (Dummy2_PhysAct excluded): R^2^ = 21.6%, adjusted R^2^ = 20.8%, F(9,867) = 26.60, p ≤ 0.01							
Constant*	2.21		1.50	2.93	0.37	0.00	
Age*	− 0.009	− 0.399	− 0.011	− 0.007	0.001	0.00	1.33
10log (BMI)*	− 0.53	− 0.14	− 0.96	− 0.08	0.23	0.02	3.58
Dummy1_PhysAct*	0.04	0.09	0.01	0.07	0.01	0.01	1.04
Dummy1_Alc	− 0.02	− 0.02	− 0.06	0.03	0.02	0.51	1.05
10log (blood pressure diastolic)	− 0.21	− 0.05	− 0.56	0.15	0.19	0.27	2.09
10log (blood pressure systolic)	0.12	0.03	− 0.23	0.44	0.17	0.49	2.00
10log (waist circumference)	0.21	0.05	− 0.27	0.72	0.26	0.41	3.79
10log (cholesterol)	− 0.10	− 0.04	− 0.26	0.07	0.08	0.25	1.15
10log (reversed WAI + 1)	− 0.03	− 0.04	− 0.08	0.02	0.02	0.23	1.08
Step 4 (10log (blood pressure systolic) excluded): R^2^ = 21.6%, adjusted R^2^ = 20.9%, F(8,868) = 29.90, p ≤ 0.01							
Constant*	2.30		1.59	2.95	0.35	0.00	
Age*	− 0.009	− 0.397	− 0.010	− 0.007	0.001	0.00	1.32
10log (BMI)*	− 0.53	− 0.14	− 0.97	− 0.09	0.23	0.02	3.58
Dummy1_PhysAct*	0.04	0.08	0.01	0.07	0.01	0.01	1.03
Dummy1_Alc	− 0.01	− 0.02	− 0.06	0.03	0.02	0.49	1.05
10log (blood pressure diastolic)	− 0.13	− 0.03	− 0.42	0.17	0.15	0.37	1.27
10log (waist circumference)	0.22	0.05	− 0.25	0.69	0.24	0.37	3.78
10log (cholesterol)	− 0.10	− 0.04	− 0.27	0.06	0.08	0.23	1.15
10log (reversed WAI + 1)	− 0.03	− 0.04	− 0.08	0.01	0.02	0.20	1.07
Step 5 (Dummy1_Alcohol excluded). R^2^ = 21.6%, adjusted R^2^ = 20.9%, F(7,869) = 34.12, p ≤ 0.01							
Constant*	2.32		1.59	3.06	0.36	0.00	
Age*	− 0.009	− 0.398	− 0.010	− 0.007	0.001	0.00	1.31
10log (BMI)*	− 0.52	− 0.13	− 0.98	− 0.10	0.23	0.02	3.56
Dummy1_PhysAct*	0.04	0.08	0.01	0.06	0.01	0.01	1.03
10log (blood pressure diastolic)	− 0.13	− 0.03	− 0.43	0.15	0.15	0.36	1.27
10log (waist circumference)	0.20	0.05	− 0.29	0.68	0.26	0.44	3.74
10log (cholesterol)	− 0.10	− 0.04	− 0.26	0.05	0.08	0.19	1.14
10log (reversed WAI + 1)	− 0.03	− 0.04	− 0.08	0.02	0.03	0.22	1.07
Step 6 (10log (waist circumference) excluded). R^2^ = 21.5%, adjusted R^2^ = 21.0%, F(6.870) = 39.72, p ≤ 0.01							
Constant*	2.49		1.96	3.06	0.28	0.00	
Age*	− 0.009	− 0.392	− 0.010	− 0.007	0.001	0.00	1.24
10log (BMI)*	− 0.38	− 0.10	− 0.63	− 0.10	0.13	0.01	1.18
Dummy1_PhysAct*	0.04	0.09	0.01	0.07	0.01	0.00	1.02
10log (blood pressure diastolic)	− 0.13	− 0.03	− 0.43	0.16	0.15	0.37	1.26
10log (cholesterol)	− 0.10	− 0.04	− 0.25	0.05	0.08	0.17	1.14
10log (reversed WAI + 1)	− 0.03	− 0.04	− 0.08	0.02	0.02	0.19	1.07
Step 7 (10log (blood pressure diastolic) excluded). R^2^ = 21.4%, adjusted R^2^ = 21.0%, F(5.871) = 47.52, p ≤ 0.01							
Constant*	2.31		1.98	2.59	0.16	0.00	
Age*	− 0.009	− 0.400	− 0.010	− 0.007	0.001	0.00	1.15
10log (BMI)*	− 0.41	− 0.10	− 0.63	− 0.16	0.12	0.00	1.08
Dummy1_PhysAct*	0.04	0.08	0.01	0.07	0.01	0.01	1.01
10log (cholesterol)	− 0.11	− 0.04	− 0.26	0.05	0.08	0.17	1.13
10log (WAI-reversed)	− 0.03	− 0.04	− 0.08	0.02	0.03	0.22	1.07
Step 8 (10log (reversed WAI + 1)) excluded). R^2^ = 21.3%, adjusted R^2^ = 20.9%, F(4.872) = 59.01, p ≤ 0.01							
Constant*	2.31		2.01	2.63	0.16	0.00	
Age*	− 0.009	− 0.406	− 0.010	− 0.007	0.001	0.00	1.12
10log (BMI)*	− 0.42	− 0.11	− 0.66	− 0.19	0.12	0.00	1.07
Dummy1_PhysAct*	0.04	0.08	0.01	0.06	0.01	0.01	1.01
10log (cholesterol)	− 0.12	− 0.05	− 0.27	0.03	0.08	0.13	1.12
Step 9 (10log (cholesterol) excluded). R^2^ = 21.1%, adjusted R^2^ = 20.8%, F(3.873) = 77.90, p ≤ 0.01							
Constant*	2.28		1.95	2.60	0.16	0.00	
Age*	− 0.009	− 0.418	− 0.011	− 0.008	0.001	0.00	1.04
10log (BMI)*	− 0.45	− 0.11	− 0.67	− 0.21	0.12	0.00	1.05
Dummy1_PhysAct*	0.04	0.08	0.01	0.07	0.01	0.01	1.01

If relevant, data are transformed to meet the assumption of a normal distribution. The 95% confidence interval around B is calculated with a bias-corrected and accelerated (BCa) bootstrap procedure (1000 samples)

*MHRR* Mean Heart Rate Range, *Unstandardized B* regression coefficient, *SE* standard error, *Standardized B* Standardized regression coefficient, *BMI* Body Mass Index, BCa bias-corrected and accelerated bootstrap, *VIF* variance inflation factor

Dummy1_PhysAct: 0 = healthy physical active behavior, 1 = moderate or unhealthy physical active behavior. Dummy2_PhysAct: 0 = healthy or moderate active behavior, 1 = unhealthy physical active behavior. Dummy1_Alc: 0 = healthy alcohol consumption, 1 = moderate or unhealthy alcohol consumption. Dummy2_Alc: 0 = healthy or moderate alcohol consumption, 1 = unhealthy alcohol consumption

*Significant: p < 0.05 and 95% BCa did not cross zero
